# A machine learning approach to assess Sustainable Development Goals food performances: The Italian case

**DOI:** 10.1371/journal.pone.0296465

**Published:** 2024-01-02

**Authors:** Tommaso Castelli, Chiara Mocenni, Giovanna Maria Dimitri

**Affiliations:** Department of Information Engineering and Mathematics, University of Siena, Siena, Italy; Nanjing University of Science and Technology, CHINA

## Abstract

In this study, we introduce an innovative application of clustering algorithms to assess and appraise Italy’s alignment with respect to the Sustainable Development Goals (SDGs), focusing on those related to climate change and the agrifood market. Specifically, we examined SDG 02: Zero Hunger, SDG 12: Responsible Consumption and Production, and SDG 13: Climate Change, to evaluate Italy’s performance in one of its most critical economic sectors. Beyond performance analysis, we administered a questionnaire to a cross-section of the Italian populace to gain deeper insights into their awareness of sustainability in everyday grocery shopping and their understanding of SDGs. Furthermore, we employed an unsupervised machine learning approach in our research to conduct a comprehensive evaluation of SDGs across European countries and position Italy relative to the others. Additionally, we conducted a detailed analysis of the responses to a newly designed questionnaire to gain a reasonable description of the population’s perspective on the research topic. A general poor performance in the SDGs indicators emerged for Italy. However, from the questionnaire results, an overall significant interest in the sustainability of the acquired products from italian citizens.

## Introduction

The agrifood market is one of the most important economic sectors for the Italian economy [1]. Such aspect is inherently entangled to climate change, and to the analysis of the climate change scenarios, and it involves attention towards sustainability issues themes which are becoming an increasing compelling research issue nowadays [2–4]. For instance [5] the Mediterranean area is considered as one of the most exposed regions, where a rise in the average temperature may prolong the duration of the growing season for the northern hemisphere benefiting crop productivity while reducing crop yields in southern located regions. Climate change may also trigger in the future extreme weather events like drought and the so called mega–drought. Such phenomena could damage crop production by affecting plant functions like photosynthesis, and heavy precipitations, which could lead to water redistribution and soil erosion. A connection between climate change and the occurrence of these extreme events is reported and clearly shown in studies as for instance [6]. It is in fact clear that the vulnerability of the agricultural sector is a matter of global concern, as it jeopardizes the production and availability of food due to irreversible shifts in weather patterns. This, in turn, poses a challenge to global food distribution, especially in countries where agriculture plays a significant role in their economy and overall productivity. Climate change also endangers the survival of many species by altering their ideal temperature ranges, leading to a progressive loss of biodiversity as ecosystem structures change. These weather variations increase the likelihood of certain diseases transmitted through food, water, and vectors, with the recent coronavirus pandemic serving as a notable example. Furthermore, climate change intensifies the mystery of antimicrobial resistance, presenting an additional threat to human health due to the rising incidence of infections resistant to treatment. [7] Due to the increase in local extreme events, and in the differences existing between geographically distant countries [6, 8] the effects of climate change are sensitive to many factors. For the above reasons, we considered a specific region of interest in our analysis, focusing mainly on the European situation, concentrating then in the positioning of the Italian region with respect to the rest of the countries considered. The European Union is in fact widely diverse and is composed of regions that will be affected in several different ways depending also on the evolution of the situation throughout the years. Moreover, we should keep in mind the fact that the relationship between food and climate change is not of the one-way type; agriculture has in fact a strong impact on the European ecosystem too [9]. For instance, the amount of land used for agriculture increases as the population increases, the emission of GreenHouse Gases (GHG) connected to agriculture and the agrifood market as a whole grows with the livestock needed to satisfy the demand for meat, dairy products and derived [10]. More specifically for what concerns Italy the effects of climate changes could become economically wise catastrophic, because of the importance of the sector in the economy of the country [11] A few of the effects of climate change into agriculture, can already be seen, in particular [5]:

- The decrease in crop water productivity in the south primarily caused by climate change.
- The decrease in cereal yield in the North and South effect of climate change too.
- The decrease in agricultural land use which is the result of urbanization, climate change and soil fertility degradation.

For these reasons, several works in the literature are present concerning performances, sustainability and the improvements that may be made to increase the efficiency and effectiveness of this sector while maintaining the same level of sustainability [12–14]. In this context the Sustainable Development Goals (SDGs) play a fundamental role. Such goals were produced as part of the 2030 Agenda for Sustainable Development, unanimously embraced by every United Nations Member State in 2015, offering a common framework for promoting harmony and well-being for both humanity and the environment, today and in the years to come. Its core comprises the 17 Sustainable Development Goals (SDGs), which represent an immediate appeal for collaboration from all nations, whether developed or developing, in a global alliance. These goals acknowledge that eradicating poverty and other forms of deprivation must be pursued alongside strategies to enhance healthcare and education, reduce disparities, foster economic progress, all while addressing the challenges of climate change and working to safeguard our oceans and forests. Full information can be found at https://sdgs.un.org/goals.

Our study presents several novelties. First of all, to the best of our knowledge, no previous studies presented an overall data analysis of SDG2, SDG12, and SDG13, looking at the European situation and comparing Italy with respect to the remaining countries. Moreover, we collected a completely novel survey, showing an inverse trend that links the interests of the Italian citizens with respect to Italy as a nation positioning with respect to the other countries. Therefore studies like ours would be beneficial for encouraging politicians and policy makers to make adequate changing also in the overall attention with respect to such themes, also from a legislative action.

The paper is structured as follows. In Section we introduced the relevant background literature for what concerns Sustainable Development Goals, as well as applications of Machine Learning in the context of SDGs analysis. In Section we will draw a description of the SDGs used in our study. In Section, together with a definition of the methodologies with which we crawled the relevant datasets from the publicly available repositories, we will give an overview of the clustering algorithm used will also be presented. In Section we will instead overlook the relevant experimental settings and results obtained, with respect to the SDGs analysed and the positioning of Italy with respect to the other European countries. Moreover in Section we will draw conclusions and future works for our analysis, focusing on the main results and possible developments described.

## Background

Sustainable Development Goals performances have been analyzed and computed since the introduction of the 2030 agenda in 2014, different approaches and different subsets of indicators have been employed to produce place lists, to understand the current situation of different countries or different sets of countries (Europe, USA, America as a whole for example). SDGs also introduced new problems to be solved and questions to be answered. In particular, for what concerns the data analysis field, some research questions concern: how to use machine learning to better understand the performances, how will Big Data impact the sustainability of countries, or how can so much data can be obtained, stored and used when needed. Performance measures of several indicators and sectors for Italy have been computed a number of times, from this point of view, the novelties of this study lie on the use of the clustering approach to understand the current and previous placement of our country with respect to the others and on the analysis of the clusters along the time period between 2000 and 2020, and the connection searched between citizens and policies (evaluated using the indicators and the performances). In the following subsections, we will briefly describe the history of Sustainable Development Goals, and later we will go through an overview of relevant studies in which a machine learning approach is used for the analysis of SDGs and their impact on society.

### Sustainable Development Goals (SDGs)

Sustainable Development Goals (SDGs) are the results of twenty years of collaborations and conferences between several countries (https://sdgs.un.org/goals). More specifically on 3–14 June 1992: the United Nations Conference on Environment and Development (UNCED), also known as ‘Earth Summit’, begins in Rio de Janeiro with the participation of political leaders, scientists, and NGOs from 179 countries to discuss the environmental situation. This summit concluded that the concept of sustainable development was possible in reality for everyone on the planet, it recognized that integrating social and environmental concerns into policy-making was essential for the planet and humans’ well-being, all these new perspectives warranted a change in the way people live, produce and consume. Among the many achievements of this conference, Agenda 21 and the Rio Declaration were the most important. Later on 6–8 September 2000: another summit was held by the United Nations in their headquarters in New York, at the time it was the largest summit of heads of state in history, and it led to the adoption of the Millennium Declaration by the 189 Members States. This declaration contained eight Millennium Development Goals (MDGs), on which the modern SDGs are based, the objectives were: extreme poverty and hunger eradication, universal primary education, promotion of gender equality and women empowerment, child mortality reduction, maternal health improvement, combat contagious diseases (i.e., HIV/AIDS), ensure environmental sustainability, development of a global partnership for development.

- June 2012: At the United Nations Conference on Sustainable Development (also known as “Rio +20”) member states launched a process to develop the Sustainable Development Goals using the MDGs as the foundation
- 2013: an Open Working Group of 30 states was established to develop the proposal
- January 2015: the negotiation process around the SDGs begins
- 25–27 September 2015: during a UN Sustainable Development Summit in New York the 2030 development agenda with the 17 newly designed SDGs is adopted by the Member States
- 12 December 2015: the Paris Agreement is signed by 196 States, the objective of this agreement is to keep the global world temperature “well” below 2°C above the pre-industrial levels and to limit the temperature increase to 1.5°C above pre-industrial levels

**Fig 1:**
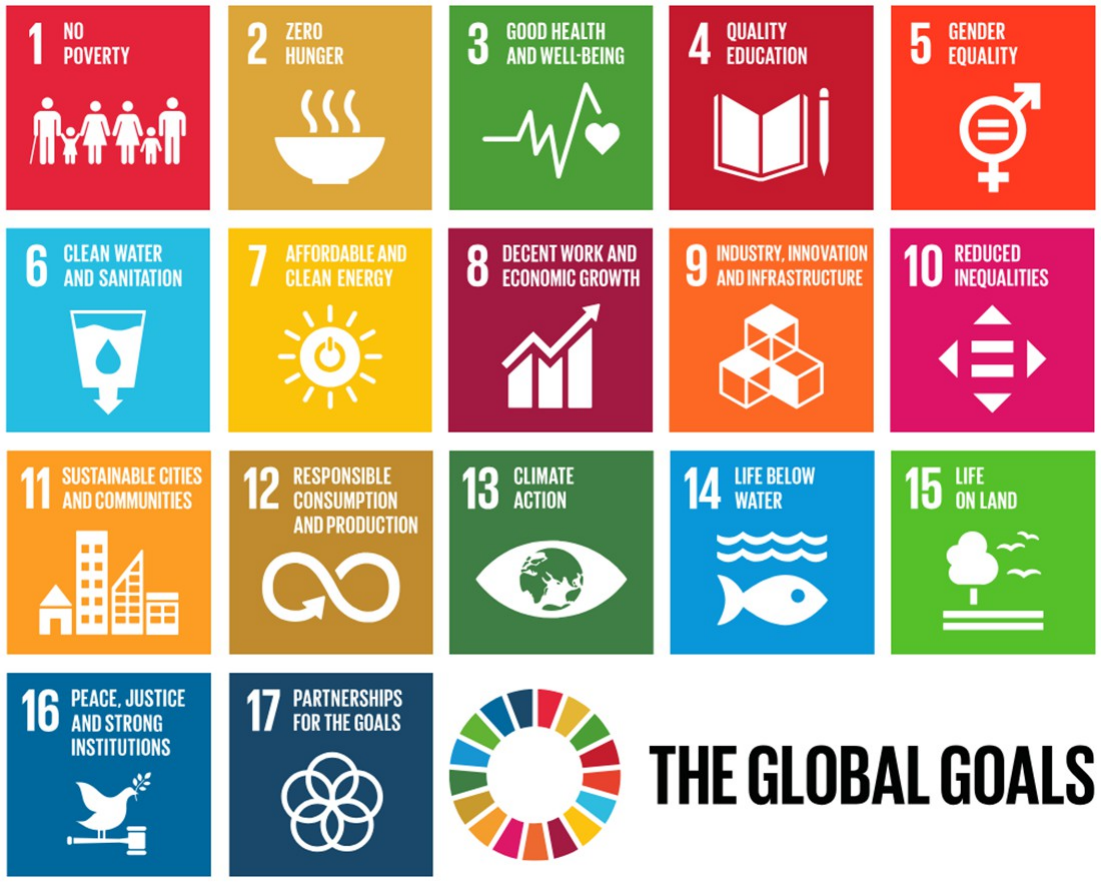
The 17 Sustainable Development Goals.

The Sustainable Development Goals adopted are 17 with a total of 169 targets to reach by 2030. All the information reported here were acquired on the official United Nations site, the complete list of all of the 17 Sustainable Development Goals can be found at https://sdgs.un.org. In Fig 1 we summarizing the 17 goals In our work we performed a selection of the SDGs, referring only the ones concerning food, i.e. SDG 02, SDG 12 and SDG13.

### Machine learning and SDGs

Machine Learning and Artificial Intelligence methods have been widely used in the literature in order to deeper the understanding of interdisciplinary and complex phenomena such as climate change, food production, bioinformatics and other complex citizen science phenomena and economics [6, 15–20]. For instance in [21], the authors studied the performances of the Italian agrifood market from a new, and very important point of view, a subset of the Sustainable Development Goals [22, 23], which were put in place by the United Nations in 2014 [24], considering the agrifood market and the sustainability aspects, and it is composed of indicators belonging to SDG02: Zero Hunger, SDG12: Responsible Consumption and Production, and SDG13: Climate Change [25]. However their study concentrated mainly on the spanish supply chain, with no use of machine learning method in the analysis proposed. Furthermore in [26] the authors designed Persephone, a machine learning model to support a network of researchers in the realization of interdisciplinary evidence syntheses in support of SDG02. In [27] the authors investigated the arable land use and poverty but did not use directly SDGs performance indicators collected in the different countries. To the best of our knowledge, no study concentrating on unsupervised clustering of the countries with respect to SDGs performances concerning food and climate was previously performed. Other works, connected the use of SDGs for the definition of climate actions as for instance [28] where the analysis of SDG12 performances are extensively reported trying to understand the achievement of the target with respect to climate change, and considering the trade offs between the target and climate actions [29]. To the best of our knowledge no previous work in which an unsupervised approach for defining the relationships between countries and sustainable development goals indicators can be found in the literature.

## Materials

In the following subsections we will first describe the datasets used in our study. Subsequently we will describe the clustering method used, in order to group the countries under examination for the purpose of gaining information on the position of Italy with respect to the rest of the European countries. All of the material used for the research is publicly accessible at: https://github.com/GiovannaMariaDimitri/SDGMachineLearning.

### Sustainable Development Goals dataset

The datasets employed for the analysis were provided by Eurostat [30] and were analysed importing them with the use of the R programming language (https://www.r-project.org/) with the Eurostat package (https://cran.r-project.org/web/packages/eurostat/index.html). We performed a preliminary selection of the SDGs included in our analysis, focusing only on those related to food and climate. In particular the ones we chose are the following (we will list their names together with their relative descriptions and subsections):

- **SDG02**: Zero hunger:
  - Agricultural factor income per annual work unit (used as a measure of the standard of living for farmers in the European Union) [SDG_02_20]
  - Area under organic farming (used as a measure of the proportion of agricultural area under productive and sustainable agriculture indicator 2.4.1 even if organic farming is not mentioned in the SDGs agenda here it is considered as the form of farming with the highest protection of the environment) [SDG_02_40]
  - Government support to agricultural research and development (used as a measure of how much priority governments place on agriculture) [SDG_02_30]
- **SDG12** Responsible consumption and production:
  - Raw material consumption (considered similar to indicator 12.2.1 “Material footprint”) [SDG_12_21]
  - Circular material use rate (considered as an indicator of adaptation of sustainable practices within the cities) [SDG_12_41]
  - Gross value added in the environmental goods and services sector (considers only the part of the economy engaged in the production of goods and services connected to the protection of the environment) [SDG_12_61]
  - Generation of waste excluding major mineral wastes by hazardousness (used as an indicator of the current situation regarding the adoption of circular economy practices which is strictly connected to SDG12) [SDG_12_50]
- **SDG13** Climate action:
  - Net greenhouse gas emission (indicator for the current situation regarding the emission of greenhouse gasses in the European Union) [SDG_13_10]
  - Net greenhouse gas emission of the Land use, Land use change and Forestry (LULUCF) (used as the previous one, an indicator of the emissions of a country) [SDG_13_21]
  - Climate-related economic losses (used to monitor the progress toward the Sustainable Development Goal, given that it aims to increase the defenses and the resilience of countries against climate-related hazards) [SDG_13_40]
  - Contribution to the international 100 billion USD commitment on climate-related expending (considered as identical to indicator 13.1: “mobilized amount of USD per year starting in 2020 accountable towards the $100 billion commitment”) [SDG_13_50]

The datasets underwent a series of preprocessing steps. We subsetted them according to years, in order to be able to better assess performances of the different European countries yearly. In this way, we were able to better define Italy’s positioning with respect to the rest of Europe in the period considered in the analysis, specifically from the year 1999 onwards.

### Dataset: The questionnaire

In this section we will further describe the structure of the questionnaire which we designed in a novel way. The reason why we decided to proceed using a survey, was that in this way we were able to gather an overall view on a sample of the population, concerning how people perceive and are aware of the overall issues of SDGs knowledge, in particular with reference to food and climate issues. The whole questionnaire is reported in S1 Appendix. The questionnaire was distributed through Google Survey. A total of 140 responses were collected. Informed consent of respondents was obtained by an agreement to a privacy statement included at the beginning of the survey. In some cases, when requested by the respondent, it was additionally obtained verbally by the distributor of the survey. No minors were included in the survey study. For this reason no additional ethics approval was needed [16]. The survey was kept opened for responses for a period of 2 months from the 23/12/2022 up until 23/02/2023.

Specifically, the questionnaire was used to get an insight on the actual knowledge of a sample of the Italian population regarding the sustainability argument with particular attention devoted to the SDGs and the indicators connected to them. Moreover, the administered questions could give us an overall idea of the sustainable practices put in place by individuals in Italy, in the context of groceries shopping. In addition, the responses from the questionnaires were also utilized as a measure to gauge how well government policies align with public commitment. This was done to ascertain whether there exists a disparity between the public’s propensity to embrace a more sustainable lifestyle and the government’s willingness to align with those aspirations. To achieve this, three types of questions were posed regarding knowledge of the SDGs. The relevant questions were structured around the interpretation of each indicator utilized in the study, without explicitly disclosing which indicators were under scrutiny. This indirect approach was adopted to ensure that respondents’ answers remained free from any apprehensive feelings about their knowledge of unfamiliar factors.

The questions targeted the level of knowledge, the habits, and the commitment of the citizens towards a more sustainable life. In this case, the questions were predominantly closed–answer, with multiple choice selection and with a strong willingness on better understanding the consumers’ behavior. In particular the questions dealt with: buying habits, feelings toward the sustainable industry as a whole, what they would like to see on the shelves of a supermarket, and what they are willing to do, spend and sacrifice in order to live a more sustainable life. Moreover the last few questions concerned demographic aspects used to segment the sample cohort and understand the population of respondents.

## Methods

In this section we will describe the methodology used for grouping similar countries and to analyse the communities detected in our work.

### Clustering algorithm: Affinity propagation

The primary goal of our paper was to gain insights into Italy’s position in comparison to other EU countries and its changes over time. To achieve this, we used a clustering algorithm for each year, assorting countries according to their similarity in SDGs performances. This approach enabled us to assess Italy’s current performance and determine if it exhibited above or below-average improvements from one year to the next.

As clustering algorithm we decided to apply Affinity Propagation (AP) [31, 32]. Such a choice was guided by the need of employing an approach that did not require the number of clusters in advance. As defined in [33], AP is: *“An algorithm that identifies exemplars among data points and forms clusters of data points around these exemplars. It operates by simultaneously considering all data point as potential exemplars and exchanging messages between data points until a good set of exemplars and clusters emerges.”* AP requires two inputs:

- s(i, k): the real-valued similarities between points which indicates the suitability of *k* of being an exemplar for *i*
- s(k, k): real number for each point *k*, representing the likelihood of becoming an exemplar, also called “preferences”, this value will influence the number of clusters obtained at the end

The messages exchanged are of two types which are called responsibility and availability.

- Responsibility[r(i, k)]: is sent from *i* to *k* and represents the cumulated evidence of exemplar suitability of data point *k*, it is a sort of competition of ownership of the possible exemplars.

In the first iteration of the algorithm the availabilities are zero, so the responsibilities are set to the input similarity between *i* and *k* minus the largest similarities between *i* and other exemplars. In the subsequent iterations the availabilities of points that belong to other exemplars will assume values below zero, this negativity will reduce the effect of some of the input similarities in the formula removing some exemplars from the competition. For *k* = *i* the responsibility is called self-responsibility and it is equal to the preferences minus the largest similarities between *i* and other exemplars, this value is an evidence of the suitability of *k* for being an exemplar

- Availability [a(i, k)]: gathers evidence from the data points on the quality of the exemplars currently selected.

Moreover the availability is set to the self-responsibility plus the sum of the positive responsibilities candidate exemplar *k* receives from other points. If the self-responsibility is negative, which means that the current exemplar is better suited, belonging to another exemplar rather than being an exemplar itself.

In particular the availability represents the accumulated evidence that *k* is an exemplar, based on the positive responsibilities sent to candidate *k* from other points. The messages are exclusively exchanged between pairs of points with known similarities so the number of computations and exchanges is limited, moreover, at any given point responsibilities and availabilities can be combined to identify the exemplars, the combination needed to identify them is:

$$\begin{array}{c} \text{argmax}(a(i,k)+r(i,k)) \end{array}$$ (1)

As for most of the algorithms the procedure can be terminated at any given point depending on the meeting of conditions such as: having reached the maximum number of iterations, after the changes in the messages fall below a threshold or after *n* iteration where no particular changes have been observed. In addition to such stopping techniques a damping factor can be employed to limit the influence of the possible oscillations.

Each iteration works recursively:

- Responsibilities update given the availabilities,
- Availabilities update given the responsibilities,
- Combination of the two results and update of the exemplars information

In the case of this study the distance measure employed was the standard distance measure employed by [34], i.e. the negative squared distance (or Euclidean distance):

### Global multiplexity matrix

Once the communities were identified using the AP algorithm, we further performed a comprehensive analysis of them, using the global multiplexity matrix index. This indicator is used to see how many times on the timespan of the specific indicator two countries are grouped in the same cluster. Such measure was of particular significance for our study as it offered a means to visually represent the connections between countries. It enabled us to observe which countries exhibited similar patterns for each indicator and to determine whether a country improved or declined in performance over the years for which indicator data was available [35]. Consequently, we could discern how often two countries were clustered together in the same group across various years.

## Results

In this Section, we will present the experimental setting and the results. To maintain a concise and comprehensible discussion, the provided plots will focus solely on two specific years: the year in which Italy was first evaluated (in the various indicators) and the most recent one available (2020). In addition to cluster the individual indicators, we also developed a customized performance metric. This additional indicator was calculated as a linear combination of the other metrics and served as an extra measure to assess the performance of different countries over the years of our analysis. We proceeded under the fundamental assumption that some indicators reflect better performance as they increase in value, while others are the opposite; their increase indicates worse performance. The clustering approach was also extended to the summary indicator, which represents the cumulative value of the indicators used in our research. This summary indicator offered another way to evaluate Italy’s performance, starting from the inception of the Sustainable Development Goals in 2014.

### Clustering experimental settings and results

In Table 1 we report the labels of the countries considered in our analysis:

**Table 1:** Countries abbreviation table.

*Country*	*Abbreviation*	*Country*	*Abbreviation*
Austria	AT	Italy	IT
Belgium	BE	Latvia	LV
Bulgaria	BG	Lithuania	LT
Croatia	HR	Luxembourg	LU
Cyprus	CY	Malta	MT
Czechia	CZ	Netherlands	NL
Denmark	DK	Poland	PL
Estonia	EE	Portugal	PT
Finland	FI	Romania	RO
France	FR	Slovakia	SK
Germany	DE	Slovenia	SI
Greece	EL	Spain	ES
Hungary	HU	Sweden	SE
Ireland	IE	European Union	EU

In our experimental setting we first clustered each of the indicators individually to analyse and understand the positioning of each country within the communities so obtained. We later considered a summary indicator. In the following subsections we will describe the results obtained for each of the cases considered, and we will study and understand the role and positioning of Italy with respect to the other countries. In particular we clustered the various SDGs separately, and later considered the clusters of countries for each SDG and for each year, and computed the Global Multiplexity Matrix, in order to gather insight on the positioning of Italy with respect to the other countries in the several years analysed and in the 4 SDGs considered.

### Sustainable Development Goal 02

**Fig 2:**
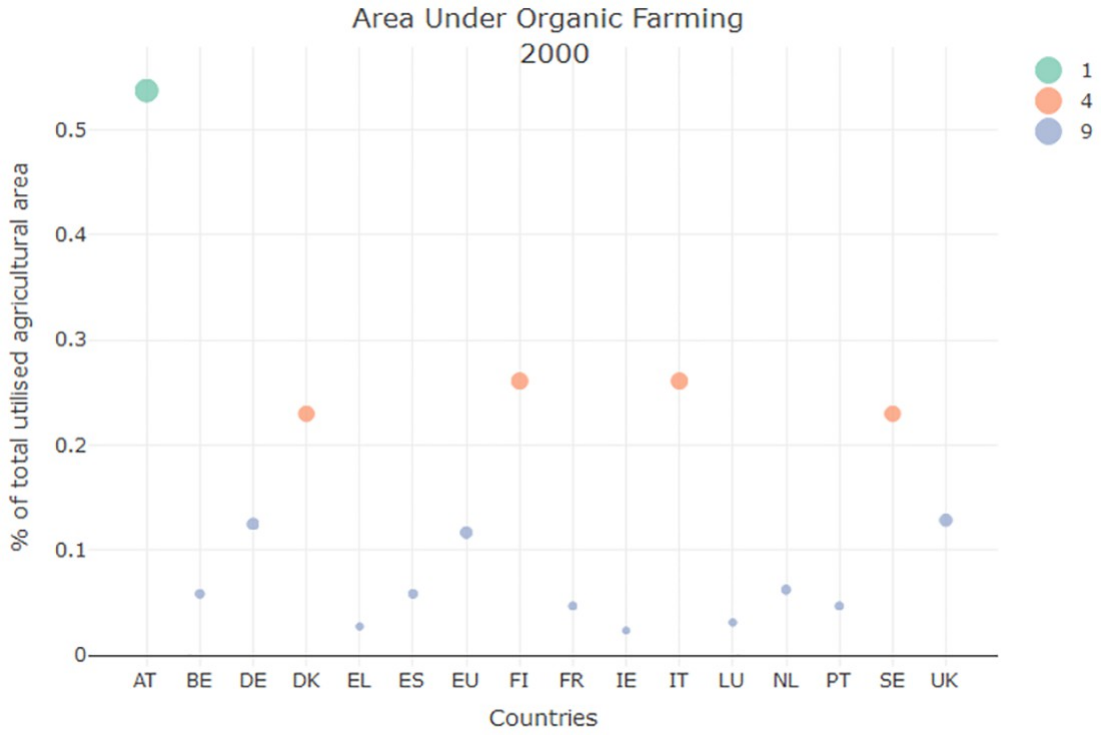
AOF clustering result for the year 2000.

SDG 02 concerns the topic of Area under organic farming (AOF). For what concerns the AOF indicator, Italy showed a strong increase (more than 100%) as can be seen in Fig 2. This improvement could be observed for the majority of the European Union countries which leads to hypothesize that this behaviour was a natural change due to the popularization of the organic food market and not an improvement due to policies with more attention towards environmental issues.

For what concerns Global Multiplexity Matrix, computing this index for SDG2 showed that Austria was the best performing country for this and for this reason it was always clustered as a singlet. Instead Italy was predominantly clustered with Finland (FI), and Czech Republic (CZ).

**Fig 3:**
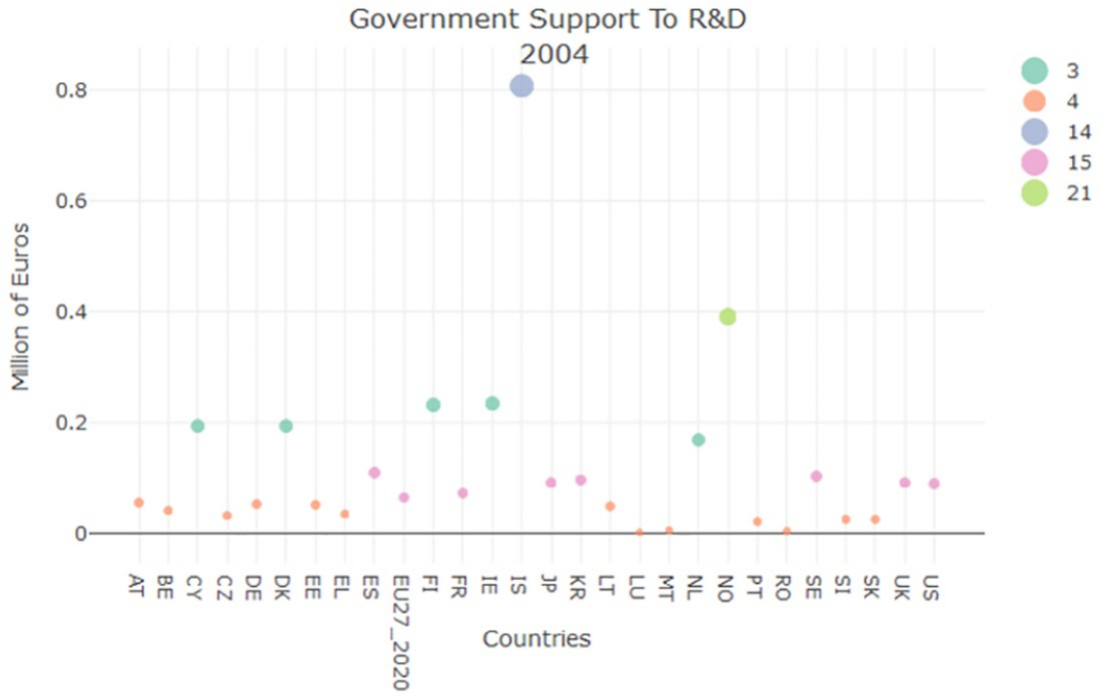
GSRD clustering result for 2005.

On the other hand for what concern the government support to Research and Development (R&D) in agriculture, Fig 3 shows a small increase in funds allocated by the country towards this sector research. If the clustering position can be considered as an approximation of a ranked list, then we could say that Italy does not perform well, always positioning itself in the lower part of the ranked list.

### Sustainable Development Goal 12

**Fig 4:**
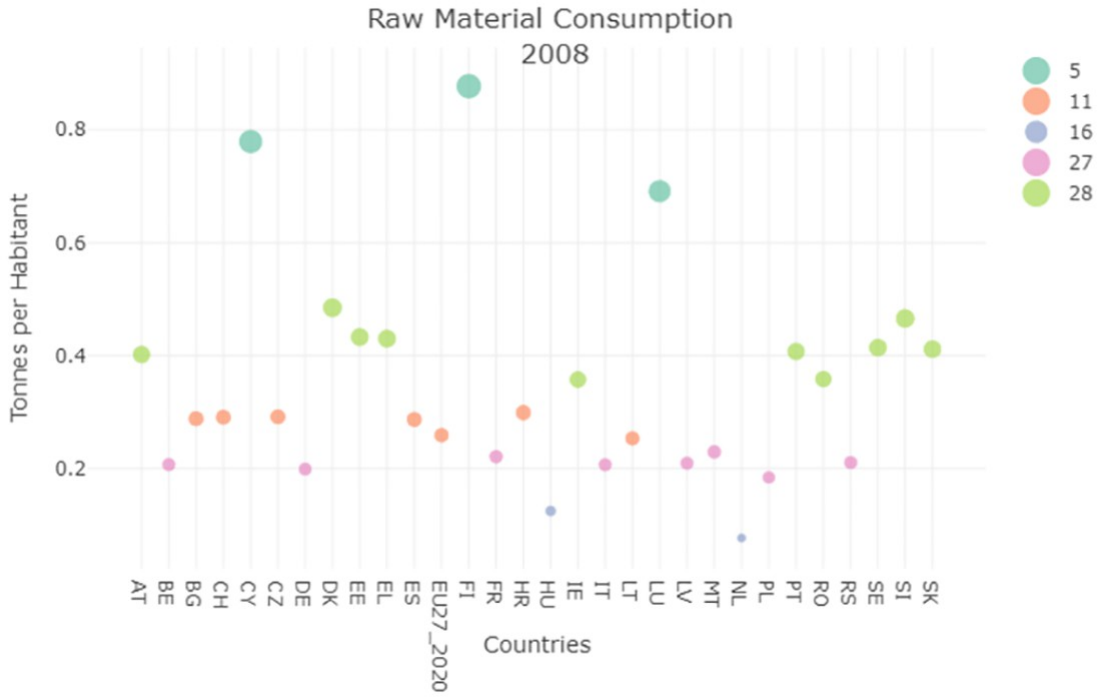
Raw material consumption clustering result 2008.

**Fig 5:**
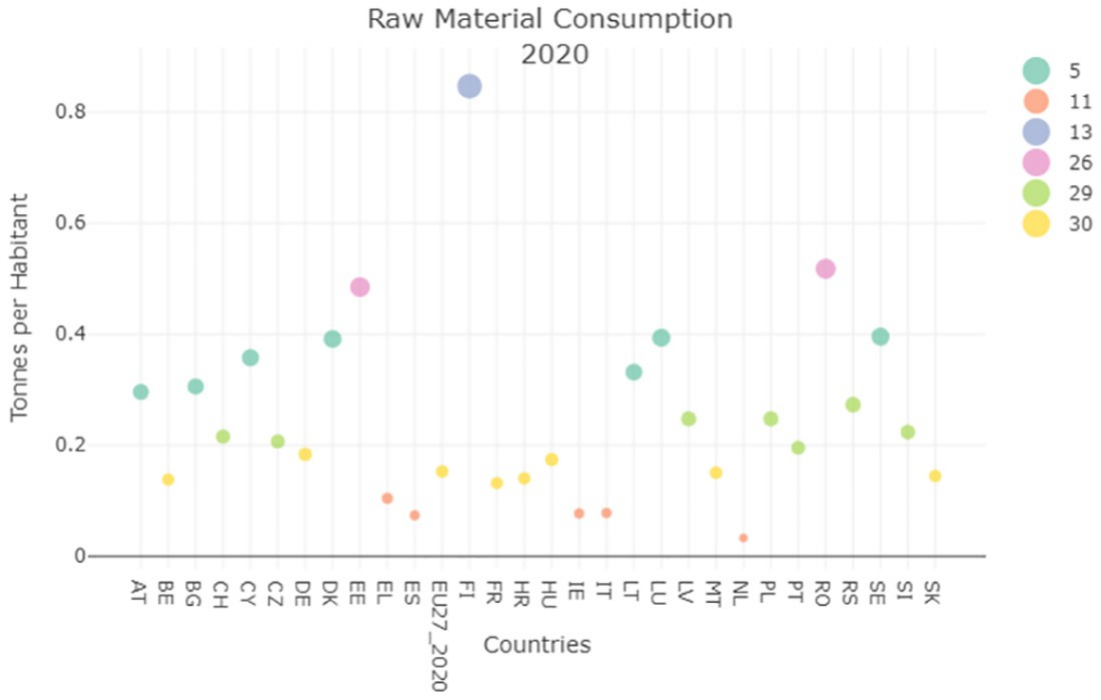
Raw material consumption clustering result 2020.

SDG12 concerns the raw material consumption indicator, this refers to the quantity of raw materials used by a country in order to produce the final product requested by the population. Therefore also in this case the smaller is the value the better are the performances of the country under examination. For what concerns the Italian situation, a decrease in the consumption recorded could be observed in Figs 4 and 5 which means that Italy improved its performances during the timespan analyzed, and places itself amongst the best performer in 2020 with a reduction of 0.05%.

**Fig 6:**
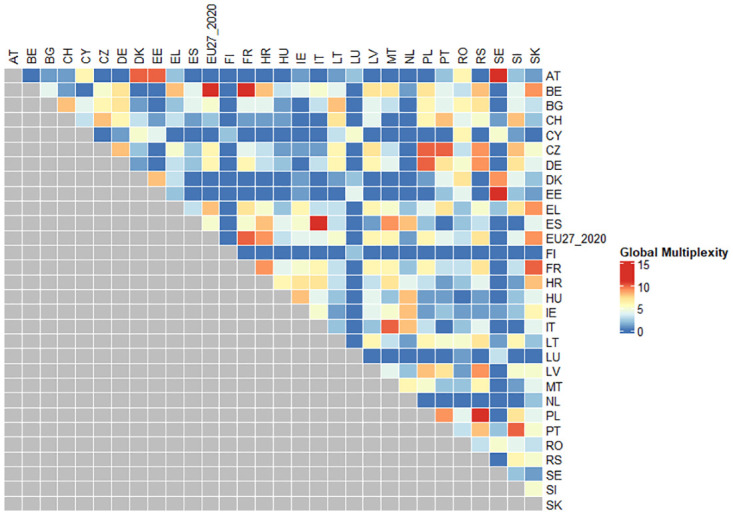
Raw material consumption global multiplexity matrix.

The global multiplexity matrix computed also showed a strong relationship between Italy (IT) and Spain (ES) as shown in Fig 6.

### Sustainable Development Goal 13

In this case this SDG concerns the contribution to the 100 billion US dollar commitment of expenditure on climate.

Such indicator uses an absolute measure to determine the performances of the different countries, not taking into account the different GDPs, looking at them for one example can help understanding how Italy behaved.

**Fig 7:**
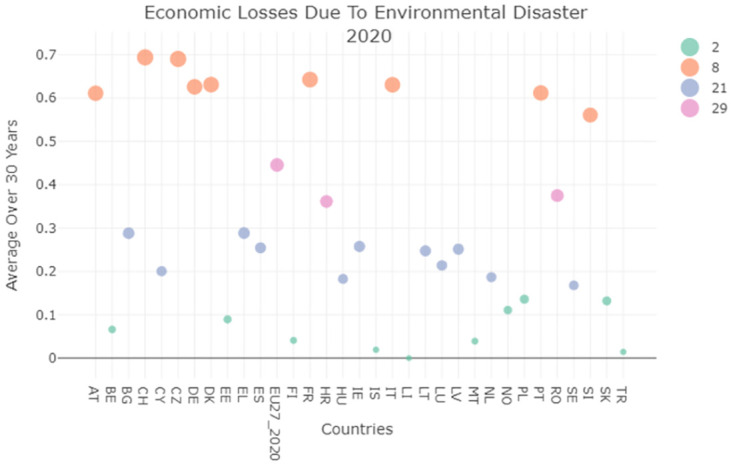
Environmental losses due to natural disasters (EL EN) average clustering result 2009.

**Fig 8:**
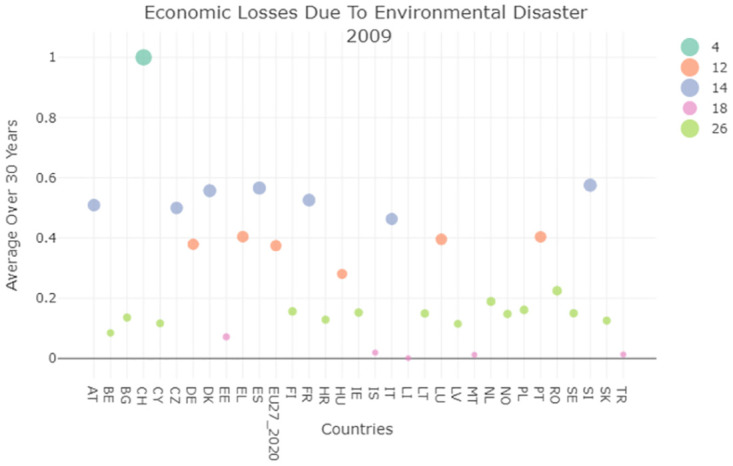
Environmental losses due to natural disasters (EL EN) average clustering result 2020.

The comparison with Germany shows that the commitment of the Italian government to support countries undergoing climate change dramatic effects, evaluated in terms of the Contribution to 100 bn USD commitment, is not comparable with other European countries. For example, Germany has a Gross Domestic Product GDP that is almost double the Italian one, this would mean that the the above indicator should be double too, instead they are 17 times higher for 2014 and 5.5 times for 2021. Such information is reported in Table 2 and was obtained from this relevant Eurostat dataset (https://ec.europa.eu/eurostat/web/sdi). We therefore also evaluated the information about environmental losses due to climate change (30 years average), also held by this SDG. This indicator is one of those indicators where better performances correspond to lower values because it is correlated to the amount of money lost due to environmental disasters. In this field, Italy is one of the worst performers as Figs 7 and 8 show. The reasons for this behavior could be related to the position and earthquake activity of Italy, but also with the lower control over constructions and the frequency with which we may have unauthorized buildings located in dangerous positions.

**Table 2:** Table representing the value of GDP per Italy and Germany respectively, considering the donations in the years 2014 and 2020.

	2014			2020		
	GDP	Contribution	%	GDP	Contribution	%
Germany	€2927 bn	€5.13 bn	∼0.17%	€3570 bn	€7.84 bn	∼0.22%
Italy	€1616 bn	€0.14 bn	∼0.01%	€1787 bn	€0.73 bn	∼0.04%

As for the generation of waste, this indicator too was used with two different units of measure: the 30 years average and the 1-year update, the average result was considered to be more comprehensible and significant with respect to the other, because of the adjustment of the average with respect to the outliers. For this indicator a lower value, implies a good performance, since it means less kgs of waste per inhabitant. Italy’s performances are worsening, passing from a value of roughly 0.2 in 2004 to 0.35 in 2020. From the global multiplexity matrix, reported in Fig 6 strong similarities can be seen among Italy, Germany and Austria, appearing always in the same clusters more than 10 times.

We further evaluated the gas emissions (tons per inhabitant), specifically the GHG indicators, contained in SDG13.

**Fig 9:**
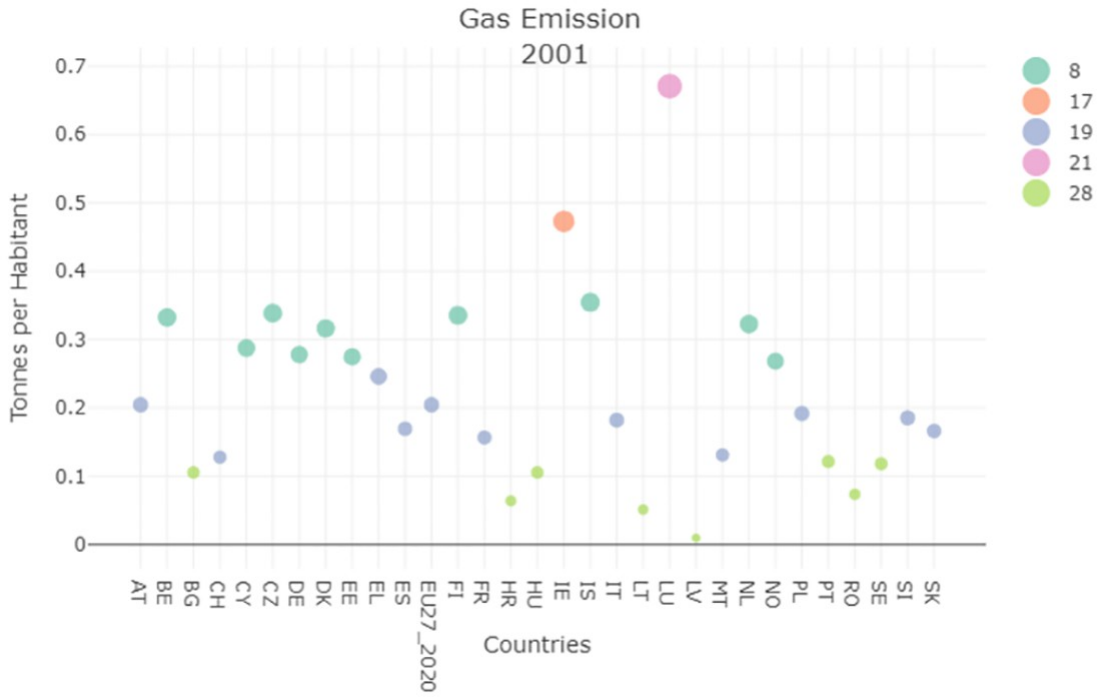
Gas emission (Tons per Habitants) clustering result 2000.

**Fig 10:**
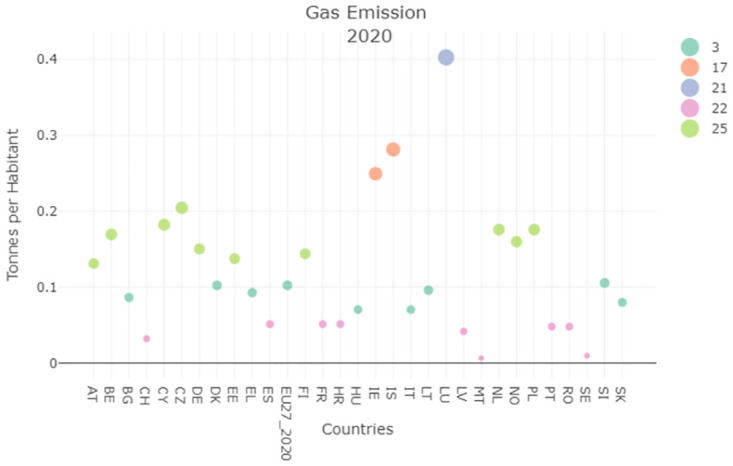
Gas emission (Tons per Habitants) clustering result 2020.

The evolution of this indicator reported by Figs 9 and 10 shows that Italy always positions itself within the lower clusters, differently from the previous unit of measure. The clustering result of this indicator shows that the Italian population emits less GHG than many EU countries (e.g., Germany, and Belgium) and that the corresponding performances improved over time.

By analysing the global multiplexity matrix, strong similarities along the years analyzed can be seen for Italy, Austria, and Spain.

### Performance function indicator

As an additional metric to study the performances and make comparisons among countries a summary indicator was computed. It takes into account the highest number of indicators that were available for all of the countries in the years 2004 to 2020. More specifically we considered: Agricultural Factor Income (AFI), Government support to R&D (GSRD), Area Under Organic Farming (AOF), Circular Material Use Rate (CMUR), Raw Material Consumption (RMC), Contribution to the 100 billion US dollar commitment of expenditure on climate (CC), GreenHouse Gases Emission (GAS), Land use change and Forestry (LULUCF) and Covenant of Mayors for climate and energies signatories (CMCE). We obtained a matrix containing in each entry the indicators’ values sum, suitably reordered in order to obtain a summary indicator where a higher value indicates better performance. Later the clustering algorithm was employed again to study the placement of Italy regarding this new indicator.

**Fig 11:**
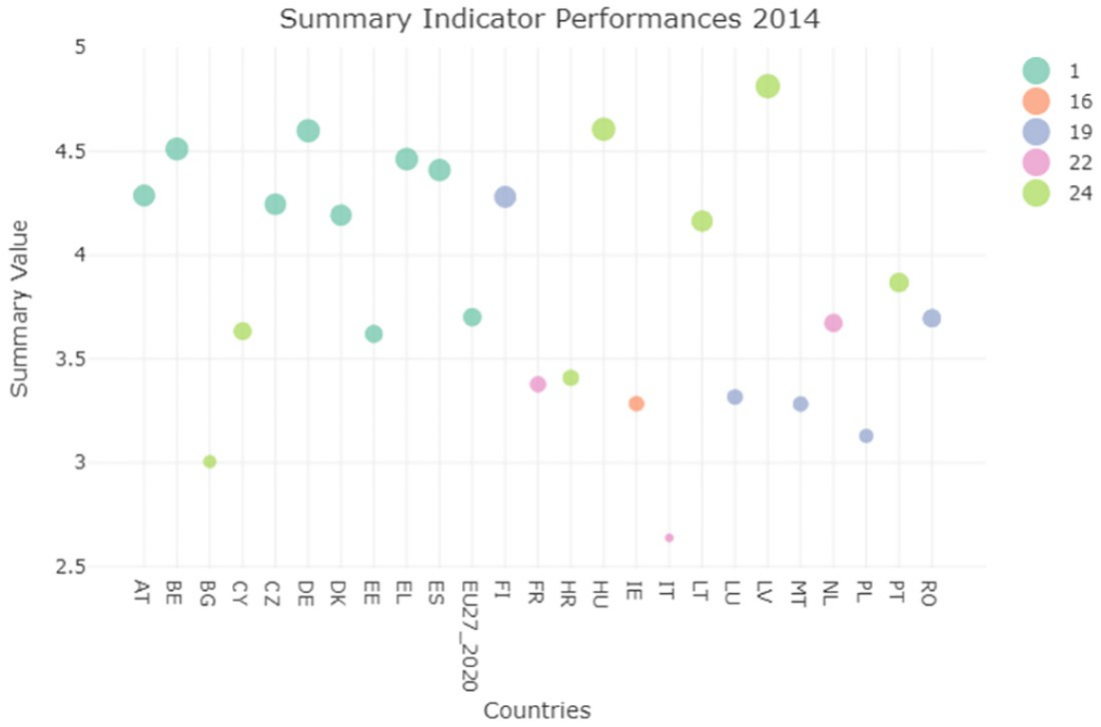
Summary plot 2014.

**Fig 12:**
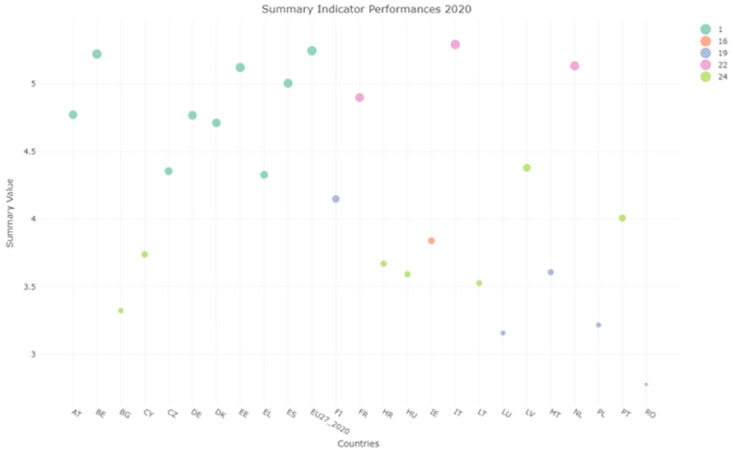
Summary plot 2020.

The clustering results for 2014 and 2020 reported in Figs 11 and 12 show an extreme variability of a lot of countries with some exceptions. From the evaluation of the clustering performances of Italy, we cannot appreciate an increasing trend throughout the years. However, a spike is present in 2020, with Italy improving its performances and resulting in the highest cluster. The spike is mainly due to the CMUR indicator, that shows a significant high performance for Italy in 2020, and therefore influencing the global performance indicator as well. Even if such spike can make us believe that the situation is improving, the goal of reaching the 2030 SDG UN goals is hard and need to be monitored carefully for Italy, in order to make sure that the proposed goals will be reached.

### Questionnaire results analysis

The questionnaire on sustainability issues was distributed anonymously to a sample of the Italian population. The total number of respondents was 140. The overall respondents were 60% women and the most frequent age range is 18–30 (50% of the respondents). Additionally the number of the respondents belonging to the 70–82, and “not declared” age class, were too few to be statistically significant and so were disregarded from the analysis. The initial question aimed at gauging the public sentiment revolved around the perceived significance of sustainability. Analyzing the response distribution, it becomes evident that individuals across all age groups recognize the immense importance of sustainability for the society, the economy, and the planet. Within each age category, more than 50% of respondents attributed the highest level of importance to social, environmental, and economic sustainability.

The second most important question for the research was connected to the knowledge regarding SDGs. The most informed subset of the population is the one aged 44–56, that, with respect to the younger groups,the 70, 6% responded ‘Yes’ to the question *‘Did you know about the existence of SDGs’* while the 18–30 age range, of which only the 55.7% knew about the goals. This could be due to the unbalance of the dataset, or to the fact that younger generations are generally more disconnected from the institutions.

A further aspect emerged from the questionnaire which is also of extreme interest: the wish, expressed by the vast majorities of respondents in all of the age groups, of being involved in the policy-making process connected to the environment and climate change. In this case all the different age ranges showed a strong desire to be more engaged in the decisions regarding these arguments, pointing to a discrepancy between the current approach to policies-making (as of now the policies are introduced by the elected government without the participation of the people) and the will of the citizens.

The questions regarding the trust in reaching some of the analyzed goals (Waste management e.g.) were used to understand population ideas towards the policies. Considering the confidence in the achievement of the goals is not high, every age range showed insecurity regarding the future, confirmed by the answer *Not sure* that is, in fact, one of the most frequent answers for these group of questions: 44% − 48%.

In order to measure the interest in the topics tackled in the research, three additional questions were asked regarding circular economy (connected to the Circular Material Use Rate (CMUR) indicator), small companies in the agri-food marker support (which can be used as a metric for the AOF indicator) and agricultural R&D (related to the Government Support to Research and Development (GSRD)). All of three answers distributions are extremely similar between each other, and the answers show that all the generations are positively interested in these topics, meaning that the common sentiment lies towards the support of circular economy as well as small companies and financing research and development to increase the sustainability of the products bought. In our questionnaire, in order to understand people’s attitudes towards responsibilities and who should bear the greatest burden, we introduced a third question on this topic. What’s noteworthy is that over half of the respondents, regardless of their age group, believe that the primary responsibility for sustainability lies with the government. This suggests that citizens perceive the most substantial changes can be achieved through the formulation and execution of policies that prioritize justice and value the well-being of our ecosystem, encompassing social, environmental, and economic aspects.

A further question was asked to understand how much thought the respondents put in the sustainability topic while shopping. In particular a direct question concerning if the respondents thought it was useful to buy sustainable products, was directed to the people interviewed. Among them 95% of the respondents said Yes, and only a very large minority responded No. Such behaviour confirmed the interests of the population towards sustainability themes, to be addressed in everyday life.

Furthermore the motivation for the shopping decisions has been investigated. The distribution of the answers to the question show that the vast majority of people that buys sustainable products do so for three main reasons:

- Environment sustainability
- Social sustainability
- Quality of the products

The answer “for the three reason at the same time” was chosen on average by 34.8% of the respondents. This means that, the customers that are actively thinking about sustainability do not think of it as a single concept, but more as a collection of practices that should improve the quality of life on earth, e.g. they are not only interested on the environment but also on the way the workers are treated. This is in line with the mission statements of SDGs that not only try to stop climate change, but also want to eliminate inequalities and injustices.

Moreover we further investigated whether consumers are satisfied with the offer of sustainable products. The results show that the customers are not very satisfied with it, so in order to better understand the reason behind this unsatisfaction, other answers were analyzed, divided by age range. The results showed that the consumers choose to buy a sustainable product because of the small impact on the environment, the low presence of chemicals, and the quality. For those who are interested in the sustainability of products, the overprice is acceptable.

The same analysis divided by age ranges gives an insight on the motifs for different ages, the younger generations put a greater importance on the sustainability, while as the respondents grow older the quality and naturality of the products become more and more important (a substantial increase of the frequency of the response “all the previous”).

**Fig 13:**
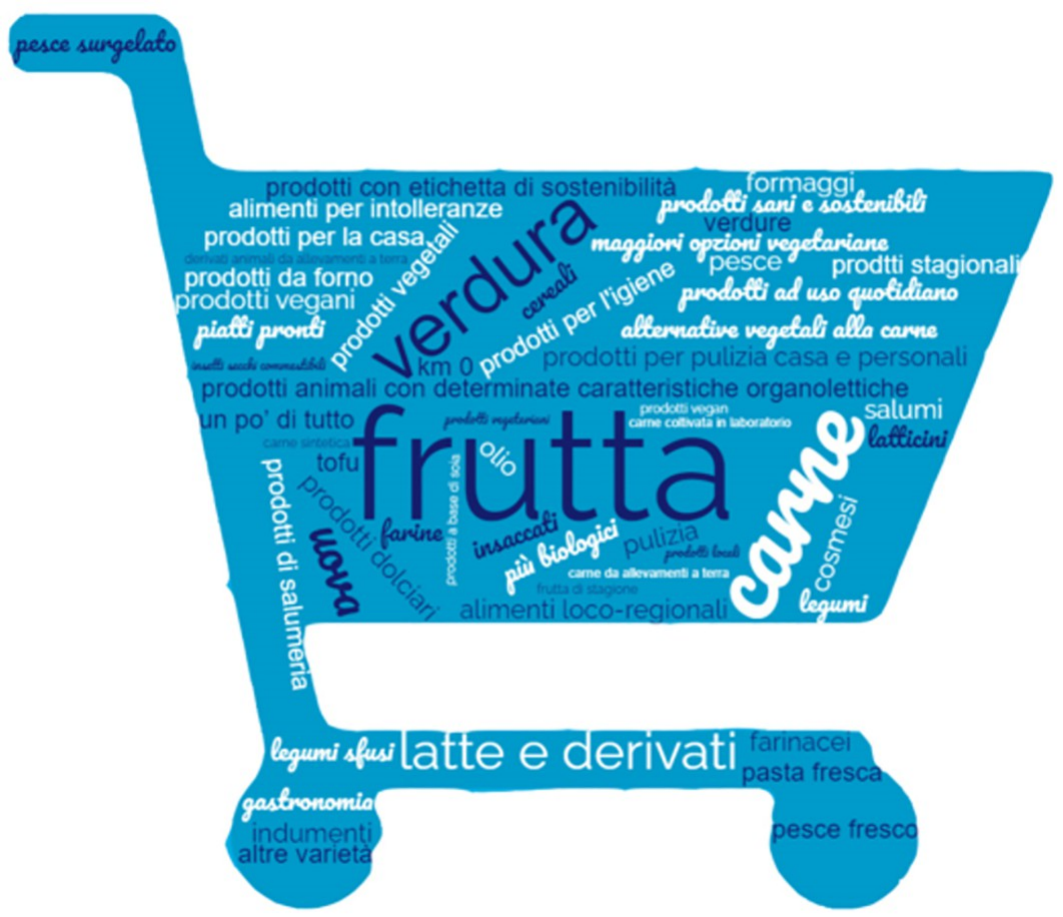
Word cloud: Most requested sustainable articles in the supermarket.

On the other hand, the vast majority (75%) of the respondents that answered *No* to question 31 felt that the high price of these products was not justified. The in-depth analysis regarding the ages of the respondents shows, once again, the difference between younger people and older ones. Specifically the latter cannot justify the increase in price, while the young feel that the small amount of money they possess could be spent on something else. Eventually, to better understand the suggestions of the respondents regarding the products that should be added to the offer of the supermarkets, a word cloud was generated and it can be seen in Fig 13.

## Conclusions

The objective of the research was to understand the positioning of Italy with respect to a sub-group of SDGs strictly connected to the agri-food market and the sustainability aspect of the economy (in particular SDG02: “zero hunger”, SDG12: “responsible production and consumption”, and SDG13: “climate action”), and then try to acquire a deeper insight on the feeling and ideas towards these arguments of the Italian population. In order to achieve both goals two approaches were employed, for the first research question the affinity propagation clustering algorithm was applied to the indicators’ datasets provided by Eurostat. The positioning of the Italian country was computed amongst the European Union countries alone and for each year. In this way the clustering result was a simple subdivision in band, with the adequate number of countries to which Italy was compared.

The second goal was achieved using a custom build questionnaire that was submitted to a sample of the Italian population, with a total of 140 respondents, the questions asked were built with three objectives in mind:

- Understand how much the population knows about the Sustainable Development Goals, and how much faith the citizen has in the achievement of these goals for 2030;
- Understand the purchase behaviors, habits, and the way of thinking of the respondents with regard to the sustainable aspect of the products bought at the supermarket or at the sustainable shop near them;
- Divide the respondents in groups using some general demographic questions to understand for example if age is a relevant divider with respect to some of the ideals and/or buying behaviors

From the experimental results, the positioning of the Italian country with respect to the vast majority of the indicators employed showed that Italy is almost always at the lower end of the ranking performance-wise, and in addition to that, the improvement in the last 20 years has not been great, in some cases it was not observable. The in-depth analysis carried out for each indicator did not show particular differences with regard to the different indicators over the years, the performances always remain mediocre, average at most, even after the introduction of the 2030 Agenda, which should have pushed the countries to do better in order to at least try to achieve some of the goals decided in 2014, instead the behavior of the Italian government did not seem to change significantly for what concerns the SDG analyzed.

The performance analysis of the combination of indicators confirmed the results of the previous analysis step, Italy exhibited mediocre performances with respect to the other countries, the most interesting thing understood from that analysis is the little to no change in the performances during the years analyzed, while the other countries, in better or worse, showed changes in the policies making approach since 2014. An exception can be found for 2020 where both the CMUR indicator and the global indicators are very high for Italy. This fact will need to be monitored in the future to confirm that it is the sign of a trend, or possibly only an outlier.

However the overall mediocre trend shown by Italy could be interpreted as a sign of little commitment from the Italian government to the implementation of more sustainable policies, a sort of block toward improvement that does not reduce the current performances. This is the main complaint on which the interviewed people agreed. It seems that this weak involvement of the government is understood by the population as an indication of low interest.

The questionnaire showed instead that the Italian population is concerned, interested, and active with regard to sustainability, and its aspects linked to what is purchased when grocery shopping. The respondents had strong opinions with regard to the responsibilities, the centrality of economic, environmental and social sustainability in today’s society, and the importance of thinking about what they are buying while shopping. This attention and involvement do not seem to be reciprocated by the government and its policies. The discrepancy between the population’s commitment to the topic and the scarce government activity regarding sustainability may indicate that, at the moment, there is a low representation within the leadership and a disconnection between the civilian needs and desires, the sciences community standing, and the policy makers. In order to reduce the differences found, a more interactive discussion between the establishment, the rest of the population and the experts should be put in place. We believe in fact, that research like ours would be ideal for establishing a connection and a discussion between the population and the politicians, and make the citizens more aware of the environmental issues and necessities to tackle food and climate change issues. Limitations of this study are represented by the availability of the indicators only for certain years, and not for the whole duration of the study considered. Further studies could in fact include a more thorough collection of data coming from different data sources, and with an overview that spans over other continents and countries. Furthermore we could increase the sample size considered in the questionnaire, to increase the sample of the population analysed and better grasp habits and understanding of the people’s knowledge and behaviour in the context of SDGs.

## Supporting information

S1 Appendix Questionnaire. (PDF)
