# Supplementary material for: A machine learning approach to assess Sustainable Development Goals food performances: The Italian case
Source: PLoS One. 2024 Jan 2;19(1):e0296465. doi: 10.1371/journal.pone.0296465 (PMC10760714; doi:10.1371/journal.pone.0296465)
Supplement: S1 Appendix — (PDF) [file pone.0296465.s001.pdf]

Appendix 1: Questionnaire

In the following the 36 questions asked in the questionnaire are reported. We will first provide the Italian version which was distributed over the sample of the population, and later we will also provide an English version of the document.

1. Sei a conoscenza dell'esistenza dei Sustainable Developement Goals (obbiettivi di sviluppo sostenibile) delle Nazioni Unite?
    a. Si
    b. No
2. Quanto ritieni importante la questione sostenibilità sociale, ambientale ed economica? Indicalo nella scala da 1 a 10

    1 2 3 4 5 6 7 8 9 10

3. Credi che la responsabilità di uno sviluppo sostenibile ricada più su:
    a. Cittadino
    b. Aziende
    c. Stato
    d. Nessuna delle precedenti
4. Indica quanto sei d'accordo con la seguente affermazione: "Realtà sostenibili ed incentrate sull'economia circolare sono essenziali per il raggiungimento di una realtà sostenibile"
    a. Molto d'accordo
    b. D'accordo
    c. Indifferente
    d. In disaccordo
    e. Molto in disaccordo
5. Vorresti essere più coinvolto nelle decisioni statali riguardanti normative sulla sostenibilità?
    a. Si
    b. No

6. Perché sì? (risposta multipla)
    a. Credo che il coinvolgimento del cittadino sia essenziale per l'implementazione di normative più giuste
    b. Credo che normative provenienti dall'alto siano scollegate dalla realtà di tutti i giorni
    c. Credo che coinvolgimento significhi anche sensibilizzazione
    d. Credo che sia un diritto/dovere di tutti partecipare al discorso sostenibilità
    e. Tutte le precedenti
7. Perché no? (risposta multipla)
    a. Credo che per queste decisioni sia necessario un livello di conoscenze che il cittadino non ha
    b. Non credo sia una problematica che influenzi il singolo cittadino
    c. Credo che prima di aumentare il coinvolgimento si debba attuare una campagna di informazione per istruire i cittadini
    d. Tutte le precedenti
8. Pensi sia possibile essere in grado di gestire e sfruttare le risorse naturali entro il 2030?
    a. Sì
    b. No
9. Indica quando sei d'accordo con la seguente affermazione: "penso che l'impegno delle multinazionali nell'adozione di pratiche sostenibili e nella sensibilizzazione in campo sostenibile sia essenziale"
    a. Molto d'accordo
    b. D'accordo
    c. Indifferente
    d. In disaccordo
    e. Molto in disaccordo
10. Pensi sia possibile ridurre in maniera sostanziale la produzione di rifiuti entro il 2030?
    a. Sì
    b. No

11. Quanto è importante per te la riduzione dello spreco di alimenti nel raggiungimento di una società sostenibile?

1 2 3 4 5 6 7 8 9 10

12. Indica quali dei seguenti obiettivi sono importanti nella battaglia al cambiamento climatico (risposta multipla)
    a. Incremento della resilienza dei paesi rispetto ai disastri ambientali
    b. Integrazione di misure legate al combattimento del cambiamento climatico nelle politiche nazionali
    c. Sensibilizzazione del cittadino
    d. Finanziamento annuale di 100 miliardi da parte di paesi sviluppati e indirizzato ai paesi in via di sviluppo per assisterli nella battaglia al cambiamento climatico
13. Indica quanto sei d'accordo con la seguente affermazione: "è importante valorizzare e sostenere le piccole imprese di produzione alimentare (agricola e non)"
    a. Molto d'accordo
    b. D'accordo
    c. Indifferente
    d. In disaccordo
    e. Molto in disaccordo
14. Quanto è importante per te l'implementazione di tecniche agricole che incrementino: la produzione per ettaro, la resilienza dell'ecosistema a disastri ecologici e la qualità dei terreni?
    a. Poco
    b. Indifferente
    c. Molto

15. Quando vai al supermercato quanto spesso pensi alla sostenibilità dei prodotti che acquisti?
    a. Mai
    b. Raramente
    c. Ogni tanto
    d. Spesso
    e. Sempre
16. Che categoria di prodotti sostenibili acquisti normalmente?
    a. Verdure
    b. Carne
    c. Latte e derivati
    d. Altro
    e. ….
17. Qual è il motivo per cui acquisti prodotti sostenibili?
    a. La sostenibilità ambientale
    b. La sostenibilità sociale
    c. La qualità dei prodotti venduti
    d. La vicinanza del negozio sostenibile a casa
    e. Tutte le precedenti
18. In una scala da 1 a 10 indica quanto è importante l'ecosostenibilità dei prodotti acquistati

    1 2 3 4 5 6 7 8 9 10

19. In una scala da uno a 10 indica quanto è importante l'estetica dei prodotti venduti

    1 2 3 4 5 6 7 8 9 10

20. Sei soddisfatto dai prodotti sostenibili presenti al supermercato?
    a. Si
    b. No
    c. Non molto

21. In caso di risposta negativa, qual è la ragione per cui non sei soddisfatto?
    a. Poca varietà di prodotti offerti
    b. Mancano dei prodotti che per me sono essenziali
    c. Il prezzo elevato dei prodotti offerti
22. Che settore secondo te ha una varietà di prodotti troppo bassa?
    a. Verdure
    b. Latte e derivati
    c. Gastronomia
    d. Altro
    e. ….
23. Che prodotti vorresti venissero aggiunti? (elenco)

24. Indica quanto sei d'accordo con la seguente affermazione "La trasparenza riguardo ai prodotti sostenibili è essenziale (provenienza, produttore, sostenibilità)"
    a. Molto d'accordo
    b. D'accordo
    c. Indifferente
    d. In disaccordo
    e. Molto in disaccordo
25. Indica quanto sei d'accordo con la seguente affermazione "I negozi sostenibili sono trasparenti riguardo alla sostenibilità e all'impatto ambientale dei loro prodotti"
    a. Molto d'accordo
    b. D'accordo
    c. Indifferente
    d. In disaccordo
    e. Molto in disaccordo

26. Che informazioni ritieni debbano essere date in aggiunta a quelle già fornite?
    a. Informazioni riguardo alla provenienza
    b. Informazioni riguardo alla sostenibilità
    c. Informazioni riguardo al produttore
27. Quale delle seguenti caratteristiche del negozio ritieni tra le più importanti? (una o più risposte selezionabili)
    a. Disponibilità di parcheggio vicino
    b. Orari di apertura
    c. Dimensione del negozio
    d. Disposizione dei prodotti
    e. Raggiungibilità a piedi o con mezzi pubblici
28. Quanto influisce la posizione del negozio nelle tue decisioni di acquisto?
    a. Poco
    b. Indifferente
    c. Molto
29. Vorresti avere più informazioni riguardo alle iniziative sostenibili e ai negozi sostenibili nella tua zona?
    a. Si
    b. No
30. Come vorresti ricevere queste informazioni?
    a. Via mail (nella newsletter)
    b. Tramite volantini
    c. Tramite i social network
31. Pensi che valga la pena di acquistare prodotti alimentari sostenibili?
    a. Sì
    b. No
32. Perché sì?
    a. Perché sono di una qualità più alta
    b. Perché hanno un basso impatto ambientale
    c. Perché sono più naturali delle loro controparti non sostenibili
    d. Tutte le precedenti

33. Perché no?
    a. Perché le qualità non giustificano un incremento del prezzo così grande
    b. Perché preferisco spendere i soldi in altri modi
    c. Perché sono molto stagionali
    d. Tutte le precedenti
34. Qual è il tuo genere?
    a. Uomo
    b. Donna
    c. Altro
    d. Preferisco non specificare
35. Quanti anni hai?
    a. 18-30
    b. 31-43
    c. 44-56
    d. 57-69
    e. 70-82
    f. Preferisco non dichiarare
36. Qual è la tua occupazione?
    a. Impiegato
    b. Libero professionista
    c. Studente
    d. Disoccupato
    e. Preferisco non specificare

English Translation

1. Are you aware of the existence of the United Nations Sustainable Development Goals?
    a. Yes
    b. No
2. How important do you consider the issue of social, environmental and economic sustainability? Indicate it on a scale of 1 to 10
    i. 12 3 4 5 6 7 8 9 10
3. Do you believe that the responsibility for sustainable development lies more on:
    a. Citizen
    b. Companies
    c. State
    d. None of the above
4. Please indicate how much you agree with the following statement: "Sustainable and circular economy-focused realities are essential for achieving a sustainable reality"
    a. Very much agree
    b. All right
    c. Indifferent
    d. Disagree
    e. Strongly disagree
5. Would you like to be more involved in state decisions regarding sustainability regulations?
    a. Yes
    b. No

6. Why yes? (multiple answer)
    a. I believe that citizen involvement is essential for the implementation of fairer regulations
    b. I believe that top-down regulations are disconnected from everyday reality
    c. I believe that involvement also means raising awareness
    d. I believe that it is everyone's right/duty to participate in the sustainability discourse
    e. All of the above
7. Why not? (multiple answer)
    a. I believe that these decisions require a level of knowledge that the citizen does not have
    b. I do not believe that this is an issue that affects the individual citizen
    c. I believe that before increasing involvement, we need to implement an information campaign to educate citizens
    d. All of the above
8. Do you think it is possible to be able to manage and exploit natural resources by 2030?
    a. Yes
    b. No
9. Indicate when you agree with the following statement: "I think that the commitment of multinational companies to adopt sustainable practices and raise awareness in the sustainable field is essential"
    a. Very much agree
    b. All right
    c. Indifferent
    d. Disagree
    e. Strongly disagree
10. Do you think it is possible to substantially reduce waste production by 2030?
    a. Yes
    b. No

11. How important is reducing food waste for you in achieving a sustainable society?

    i. 12 3 4 5 6 7 8 9 10

12. Indicate which of the following goals are important in the battle against climate change (multiple answer)
    a. Increasing countries' resilience to environmental disasters
    b. Integration of measures related to the fight against climate change into national policies
    c. Raising public awareness
    d. Annual funding of €100 billion from developed countries to assist developing countries in the fight against climate change
13. Please indicate how much you agree with the following statement: "it is important to value and support small food production enterprises (agricultural and non-agricultural)"
    a. Very much agree
    b. All right
    c. Indifferent
    d. Disagree
    e. Strongly disagree
14. How important is it for you to implement agricultural techniques that increase: production per hectare, the resilience of the ecosystem to ecological disasters and soil quality?
    a. Little
    b. Indifferent
    c. A lot

15. When you go to the supermarket, how often do you think about the sustainability of the products you buy?
    a. Never
    b. Rarely
    c. Occasionally
    d. Often
    e. All the time
16. What category of sustainable products do you normally buy?
    a. Vegetables
    b. Meat
    c. Milk and dairy products
    d. Other
    e. ....
17. What is the reason why you buy sustainable products?
    a. Environmental sustainability
    b. Social sustainability
    c. The quality of the products sold
    d. The proximity of the sustainable store to the home
    e. All of the above
18. On a scale of 1 to 10, it indicates how important the eco-sustainability of the products purchased is

    i. 12 3 4 5 6 7 8 9 10

19. On a scale of one to 10, it indicates how important the aesthetics of the products sold are

    i. 12 3 4 5 6 7 8 9 10

20. Are you satisfied with the sustainable products in the supermarket?
    a. Yes
    b. No
    c. Not much

21. If not, what is the reason why you are not satisfied?
    a. Little variety of products offered
    b. They are missing products that are essential for me
    c. The high price of the products offered
22. Which industry do you think has too little product variety?
    a. Vegetables
    b. Milk and dairy products
    c. Gastronomy
    d. Other
    e. ....
23. What products would you like to be added? (list)

24. Please indicate how much you agree with the following statement "Transparency regarding sustainable products is essential (provenance, manufacturer, sustainability)"
    a. Very much agree
    b. All right
    c. Indifferent
    d. Disagree
    e. Strongly disagree
25. Please indicate how much you agree with the following statement: "Sustainable stores are transparent about the sustainability and environmental impact of their products"
    a. Very much agree
    b. All right
    c. Indifferent
    d. Disagree
    e. Strongly disagree

26. What information do you think should be given in addition to what has already been provided?
    a. Information about the origin
    b. Sustainability information
    c. Manufacturer Information
27. Which of the following store features do you consider to be among the most important? (one or more selectable answers)
    a. Parking available nearby
    b. Opening hours
    c. Size of the store
    d. Product Arrangement
    e. Reachable on foot or by public transport
28. How much does the location of the store influence your purchasing decisions?
    a. Little
    b. Indifferent
    c. A lot
29. Would you like to learn more about sustainable initiatives and sustainable stores in your area?
    a. Yes
    b. No
30. How would you like to receive this information?
    a. By email (in the newsletter)
    b. Via flyers
    c. Via social networks
31. Do you think it's worth buying sustainable food products?
    a. Yes
    b. No
32. Why yes?
    a. Because they are of a higher quality
    b. Because they have a low environmental impact
    c. Because they are more natural than their unsustainable counterparts
    d. All of the above

33. Why not?
    a. Why the qualities do not justify such a large price increase
    b. Why I prefer to spend money in other ways
    c. Because they are very seasonal
    d. All of the above
34. What's your gender?
    a. Man
    b. Woman
    c. Other
    d. I prefer not to specify
35. How old are you?
    a. 18-30
    b. 31-43
    c. 44-56
    d. 57-69
    e. 70-82
    f. I prefer not to declare
36. What is your occupation?
    a. Employee
    b. Freelancer
    c. Student
    d. Unemployed
    e. I prefer not to specify
